# Three *ADIPOR1* Polymorphisms and Cancer Risk: A Meta-Analysis of Case-Control Studies

**DOI:** 10.1371/journal.pone.0127253

**Published:** 2015-06-05

**Authors:** Jiaxiang Ye, Li Jiang, Changliang Wu, Aiqun Liu, Sufei Mao, Lianying Ge

**Affiliations:** 1 Department of Medical Oncology, the Cancer Institute, Affiliated Tumor Hospital of Guangxi Medical University, Nanning, Guangxi 530021, P.R. China; 2 Graduate School of Guangxi Medical University, Nanning, Guangxi 530021, P.R. China; 3 Department of Gastroenterology, the First Affiliated Hospital of Guangxi Medical University, Nanning, Guangxi 530021, P.R. China; Children's National Medical Center, Washington, UNITED STATES

## Abstract

**Background:**

Studies have come to conflicting conclusions about whether polymorphisms in the adiponectin receptor 1 gene (*ADIPOR1*) are associated with cancer risk. To help resolve this question, we meta-analyzed case-control studies in the literature.

**Methods:**

PubMed, EMBASE, Cochrane Library, the Chinese Biological Medical Database and the Chinese National Knowledge Infrastructure Database were systematically searched to identify all case-control studies published through February 2015 examining any *ADIPOR1* polymorphisms and risk of any type of cancer. Pooled odds ratios (ORs) and corresponding 95% confidence intervals (CIs) were calculated.

**Results:**

A total of 13 case-control studies involving 5,750 cases and 6,762 controls were analyzed. Analysis of the entire study population revealed a significant association between rs1342387(G/A) and overall cancer risk using a homozygous model (OR 0.82, 95%CI 0.72 to 0.94), heterozygous model (OR 0.84, 95%CI 0.76 to 0.93), dominant model (OR 0.85, 95%CI 0.75 to 0.97) and allele contrast model (OR 0.88, 95%CI 0.80 to 0.97). However, subgroup analysis showed that this association was significant only for Asians in the case of colorectal cancer. No significant associations were found between rs12733285(C/T) or rs7539542(C/G) and cancer risk, either in analyses of the entire study population or in analyses of subgroups.

**Conclusions:**

Our meta-analysis suggests that the *ADIPOR1* rs1342387(G/A) polymorphism, but not rs12733285(C/T) or rs7539542(C/G), may be associated with cancer risk, especially risk of colorectal cancer in Asians. Large, well-designed studies are needed to verify our findings.

## Introduction

Cancer remains a frequent cause of death worldwide [1]. The prevalence of cancer around the world reflects, in part, the prevalence of obesity, which has been rising in parallel with living standards, not only in developed countries but also in some developing ones. Large epidemiological studies have revealed a significant association of obesity with various kinds of cancers, including colorectal, breast, endometrial, renal, esophageal, pancreatic, and biliary [2–4].

One link between obesity and cancer may be adiponectin, one of several cytokines secreted primarily by adipose tissue. Several studies suggest that adiponectin protects against obesity-related malignancy, such that higher serum levels are associated with lower risk of cancer [5–7]. Circulating adiponectin levels are influenced primarily by the activity of adiponectin receptors 1 and 2 (ADIPOR1, ADIPOR2) [8], and some studies have linked ADIPOR1 dysfunction with development of cancer [9, 10]. Exactly how the function or dysfunction of these receptors can lead to cancer remains poorly understood.

The *ADIPOR1* gene has >28 single-nucleotide polymorphisms (SNPs) and two linkage disequilibrium blocks. Several of these polymorphisms have been associated with cancer risk, but studies have reported contrasting results depending on the cancer type or population involved. Some work has concluded that certain ADIPOR1 variants, including rs1342387(G/A), protect against colorectal cancer [11, 12], whereas a third study found that rs1342387(G/A) increases the risk of this cancer [13]. A case-control study reported that several *ADIPOR1* SNPs were associated with prostate cancer risk [14], while two studies found no such association [10, 15]. One study showed no relationship between *ADIPOR1* variants and breast cancer risk [16], whereas another study concluded that the SNP rs7539542 was associated with decreased breast cancer risk [17].

To help resolve these conflicting results using as large a sample as possible, we conducted a meta-analysis of case-control studies analyzing potential associations between various *ADIPOR1* SNPs and risk of various types of cancer. We focused on the SNPs that have been studied most extensively: -1472C→T in intron 1 in linkage disequilibrium block 1 [rs12733285(C/T)], +5843G→A in intron 4 in block 1 [rs1342387(G/A)] and +10225 C→G in exon 8 in block 2 [rs7539542(C/G)].

## Materials and Methods

### Literature search

A comprehensive search was carried out using PubMed, EMBASE, Cochrane Library, the Chinese Biological Medical database and the Chinese National Knowledge Infrastructure database to identify case–control studies that were published through Feb.28, 2015 and that examined the association of *ADIPOR1* polymorphisms with cancer risk. Searches were carried out using various combinations of customized terms and the MeSH-indexed terms “adiponectin”, “*ADIPOR1*”, “polymorphism”, and “cancer”, without restrictions on publication language. The following sequential search strategy was applied for each database: (#1)'Adiponectin': ab, ti OR 'ADIPOQ': ab, ti OR 'ADIPOR1': ab, ti OR 'Adiponectin'/exp OR 'Adiponectin receptor 1'/exp; (#2)'variation': ab, ti OR 'polymorphism': ab, ti OR 'SNP': ab, ti OR 'genetic polymorphism'/exp OR 'genetic variability'/exp; (#3)'neoplasm': ab, ti OR 'cancer': ab, ti OR 'carcinoma': ab, ti OR 'tumor': ab, ti OR 'neoplasm'/exp OR 'carcinoma'/exp; (#4) #1 AND #2 AND #3. Search strings were adjusted accordingly for the other databases. References cited in identified articles were searched manually to find additional studies.

### Study inclusion and exclusion

Inclusion and exclusion criteria were established before searching the literature. To be included in our meta-analysis, studies had to (1) apply a case-control design, (2) analyze the relationship between *ADIPOR1* polymorphisms and cancer risk, and (3) report genotype data for cases and controls in sufficient detail for extracting and pooling with data from other studies. Studies were excluded if they were case reports, review articles or duplicate publications.

### Data extraction

Two investigators (JXY, LJ) independently extracted the following data from included studies: first author’s name, year of publication, country/region and ethnicity of study population, type of cancer, source of controls (population- or hospital-based), genotyping method, number of case and control genotypes, and results of Hardy–Weinberg equilibrium (HWE) testing for genotype data from the control group. If HWE results were not reported, we determined them ourselves using a web-based program (http://ihg.gsf.de/cgi-bin/hw/hwa1.pl). If other data were missing, we contacted study authors to request them.

### Quality assessment

The quality of all eligible studies was evaluated using the Newcastle–Ottawa Scale (NOS), widely used for case-control studies [18]. The NOS provides a quality rating based on criteria covering three study dimensions: study group selection, comparability of cases and controls, and exposure of cases and controls. If all criteria are met, nine stars are rewarded. Seven stars are considered the cut-off for distinguishing “high-quality studies” from “low-quality studies”.

### Statistical analysis

Odds ratios (ORs) with corresponding 95% confidence intervals (CIs) were calculated using RevMan 5.1.0 (The Cochrane Collaboration, Oxford, UK) to assess the strength of associations of *ADIPOR1* SNPs rs12733285(C/T), rs1342387(G/A) and rs7539542(C/G) with cancer risk. ORs were calculated using five genetic models: homozygous, heterozygous, dominant, recessive, and allele contrast. Subgroup analysis was also conducted according to ethnicity or cancer type. If only one study covering a particular cancer was included in the meta-analysis, we planned to categorize that study among those classified as dealing with "other" cancers.

Heterogeneity among studies was assessed using the Q-test and I² statistics. When homogeneity was considered significant (P_heterogeneity_≥ 0.1), a fixed-effect model was used; otherwise, a random-effect model was used (P_heterogeneity_<0.1). Sensitivity analysis omitting one study at a time was also performed to confirm the main source of heterogeneity. Funnel plots were visually inspected for asymmetry to estimate the potential for publication bias [19]. In order to supplement funnel plot analysis, we performed Begg’s test [20] and Egger’s test [21] using Stata 12.0 (Stata Corporation, College Station, TX).

## Results

### Study selection and characteristics

**Fig 1:**
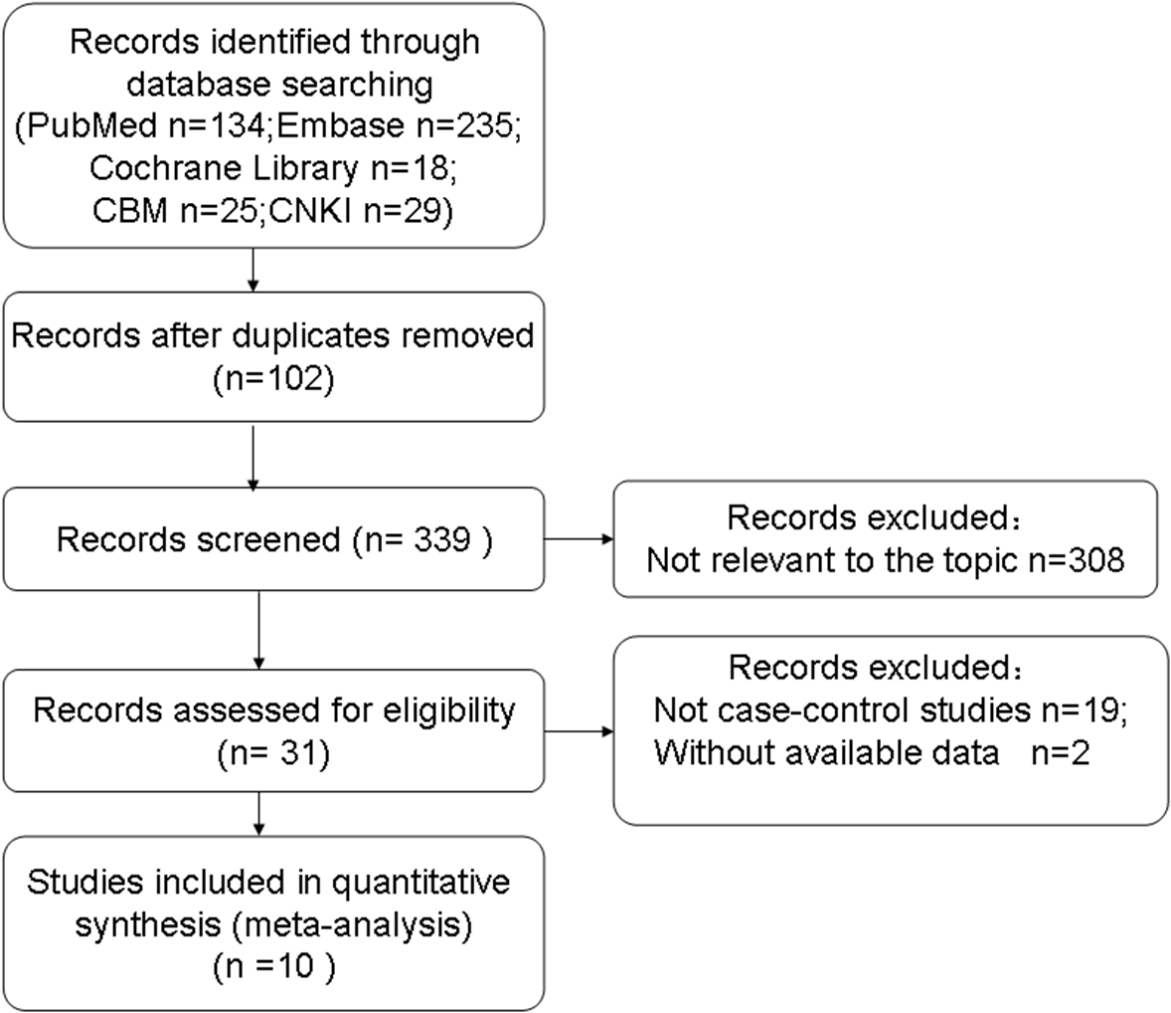
Flow diagram of study selection for the meta-analysis. CBM, Chinese Biological Medical Database. CNKI, Chinese National Knowledge Infrastructure Database.

This meta-analysis was conducted according to the recommendations of the “Preferred Reporting Items for Systematic Reviews and Meta-Analyses” (PRISMA) statement (**S1 Checklist**) and “Meta-analysis on Genetic Association Studies” statement (**S2 Checklist**). Systematic literature searches identified 10 publications [10–17, 22, 23] describing 13 case-control studies (**Fig 1**). One publication [13] described two case–control studies, and another [23] reported three case–control studies. Of the 13 studies, 10 analyzed the *ADIPOR1* SNP rs12733285(C/T) [10–14, 17, 23]; 12 analyzed rs1342387(G/A) [11–17, 22, 23]; and 8 analyzed rs7539542(C/G) [10, 13–17, 22] (**Table 1**). Genotype distribution in control groups was consistent with HWE in all 13 studies. All but two studies [15, 23] received at least seven stars on the NOS, indicating that they were high-quality (**Table 1, S1 Table**).

**Table 1:** Characteristics of studies included in the meta-analysis.

Study	Country	Ethnicity	Tumor type	Source of control	Genotyping method	Genotype data of case/control			HWE	NOS scores
rs12733285C/T						TT	CT	CC		
Dhillon 2011[10]	America	Non-Asian	PC	PB	MALDI-TOF	139/118	547/528	562/577	Yes	9
He 2011[11]	China	Asian	CRC	HB	PCR-RFLP	0/0	34/78	386/477	Yes	7
Kaklamani 2008–1[13]	America	Non-Asian	CRC	PB	Taqman	69/105	221/347	147/200	Yes	8
Kaklamani 2008–2[13]	America	Non-Asian	CRC	HB	Taqman	19/14	78/77	98/101	Yes	8
Kaklamani 2008–3[17]	America	Non-Asian	BC	PB	Taqman	100/126	294/315	321/366	Yes	7
Kaklamani 2011[14]	America	Non-Asian	PC	PB	Taqman	48/71	221/222	183/145	Yes	8
Ou 2012–1[23]	China	Asian	CRC	HB	Taqman	2/7	47/93	289/614	Yes	6
Ou 2012–2[23]	China	Asian	GC	PB	Taqman	0/0	19/15	113/121	Yes	8
Ou 2012–3[23]	China	Asian	HC	PB	Taqman	0/0	12/14	94/94	Yes	8
Zhang 2012[12]	China	Asian	CRC	HB	PCR-RFLP	0/0	30/50	340/320	Yes	7
**rs1342387G/A**						**AA**	**AG**	**GG**		
Beebe-Dimmer 2010[15]	America	Non-Asian	PC	PB	Taqman	31/74	59/172	41/87	Yes	6
He2011[11]	China	Asian	CRC	HB	PCR-RFLP	50/ 82	157/263	213/210	Yes	7
Kaklamani 2008–1[13]	America	Non-Asian	CRC	PB	Taqman	99/179	223/313	113/155	Yes	8
Kaklamani 2008–2[13]	America	Non-Asian	CRC	HB	Taqman	32/ 32	101/99	57/61	Yes	8
Kaklamani 2008–3[17]	America	Non-Asian	BC	PB	Taqman	201/209	362/419	145/180	Yes	7
Kaklamani 2011[14]	America	Non-Asian	PC	PB	Taqman	112/122	218/209	116107	Yes	8
Liu 2011[22]	China	Asian	CRC	HB	MALDI-TOF	56 /64	222/227	189/165	Yes	7
Ou 2012–1[23]	China	Asian	CRC	HB	Taqman	37 /112	135/312	159/289	Yes	6
Ou 2012–2[23]	China	Asian	GC	PB	Taqman	19 /17	57/59	59/53	Yes	8
Ou 2012–3[23]	China	Asian	HC	PB	Taqman	16/ 14	46/49	43/44	Yes	8
Teras 2009 [16]	America	Non-Asian	BC	PB	Sequencing	458/457^a^		172/184	Yes	8
Zhang 2012[12]	China	Asian	CRC	HB	PCR-RFLP	46 /58	144/172	180/140	Yes	7
**rs7539542C/G**						**GG**	**CG**	**CC**		
Beebe-Dimmer 2010[15]	America	Non-Asian	PC	PB	Taqman	54/140	56/133	19/49	Yes	6
Dhillon 2011[10]	America	Non-Asian	PC	PB	MALDI-TOF	538/543	513/489	135/135	Yes	9
Kaklamani 2008–1[13]	America	Non-Asian	CRC	PB	Taqman	44/63	209/280	179/306	Yes	8
Kaklamani 2008–2[13]	America	Non-Asian	CRC	HB	Taqman	26/24	75/81	96/89	Yes	8
Kaklamani 2008–3[17]	America	Non-Asian	BC	PB	Taqman	117/111	308/361	297/334	Yes	7
Kaklamani 2011[14]	America	Non-Asian	PC	PB	Taqman	43/45	226/193	183/194	Yes	8
Liu 2011[22]	China	Asian	CRC	HB	MALDI-TOF	172/180	219/218	78/60	Yes	7
Teras 2009[16]	America	Non-Asian	BC	PB	Sequencing	356/356^b^		281/296	Yes	8

Notes: BC, breast cancer; CRC, colorectal cancer; GC, gastric cancer; PC, prostate cancer; HC, hepatic carcinoma; PB, population-based; HB, hospital-based; MALDI-TOF matrix-assisted laser desorption/ionization time-of-flight mass spectrometry; PCR-RFLP, polymerase chain reaction-restriction fragment length polymorphism; HWE, Hardy-Weinberg equilibrium; NOS, Newcastle–Ottawa Scale.

^a^The sum of genotypes AA and AG.

^b^The sum of genotypes GG and CG.

### Quantitative synthesis

**Fig 2:**
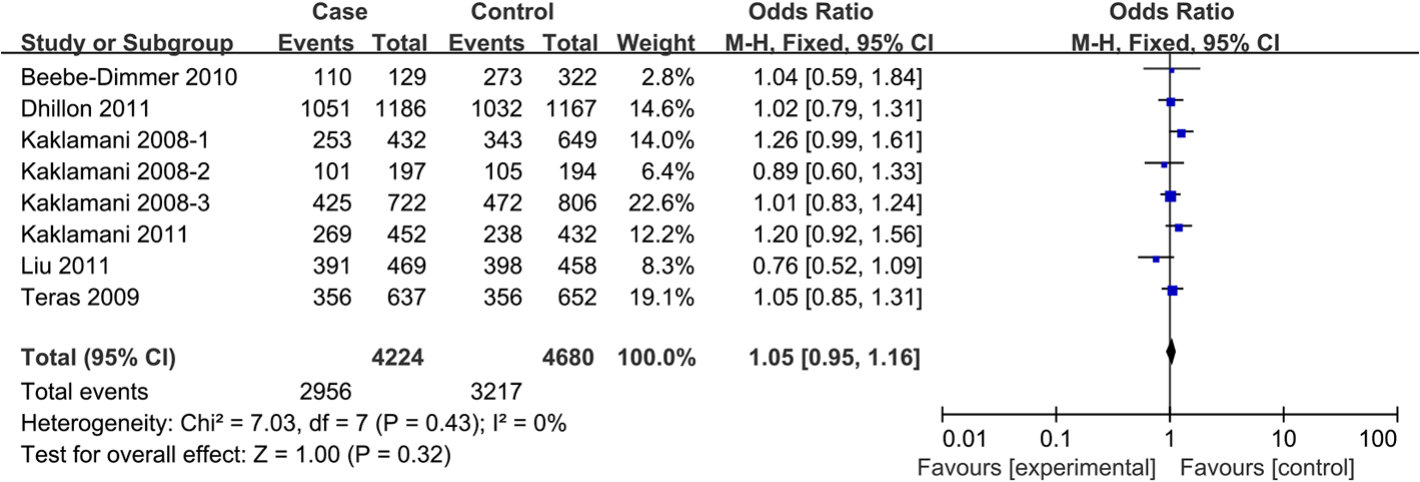
Forest plot of the association between *ADIPOR1* SNP rs7539542(C/G) and cancer risk in a dominant model.

In pooled analysis using data from all 10 studies [10–14, 17, 23], no significant association was observed between the *ADIPOR1* rs12733285C/T polymorphism and risk of any cancer, based on any of the five genetic models. Similar results were obtained in subgroup analyses (**Table 2**). A similar lack of association was observed for the SNP rs7539542(C/G) across all eight studies [10, 13–17, 22] and subgroups (**Table 3, Fig 2**).

**Table 2:** Overall and subgroup analysis of the *ADIPOR1* rs12733285(C/T) polymorphism and cancer risk.

Variable	Homozygous model		Heterozygous model		Dominant model		Recessive model		Allele contrast model	
	OR [95%CI]	P^a^	OR [95%CI]	P^a^	OR [95%CI]	P^a^	OR [95%CI]	P^a^	OR [95%CI]	P^a^
Total	0.91[0.69,1.19]	0.04	0.90[0.77,1.05]	0.04	0.89[0.76,1.04]	0.01	0.95[0.82,1.10]	0.11	0.91[0.80,1.04]	<0.1
*Ethnicity*										
Non-Asian	0.92[0.69,1.22]	0.02	0.99[0.89,1.10]	0.34	0.98[0.89,1.09]	0.11	0.94[0.75,1.19]	0.07	0.96[0.85,1.09]	0.03
Asian	0.61[0.13,2.94]	NA	0.79[0.55,1.14]	0.05	0.79[0.55,1.12]	0.05	0.60[0.12,2.91]	NA	0.79[0.57,1.09]	0.08
*Tumor type*										
CRC	0.96[0.69,1.32]	0.48	0.80[0.61,1.04]	0.05	0.81[0.62,1.05]	0.04	1.01[0.76,1.36]	0.56	0.84[0.67,1.07]	0.03
PC	0.82[0.37,1.82]	<0.1	0.94[0.70,1.25]	0.08	0.90[0.61,1.34]	0.01	0.86[0.46,1.63]	<0.1	0.92[0.65,1.30]	<0.1
Others	0.90[0.67,1.22]	NA	1.07[0.87,1.31]	0.71	0.89[0.63,1.26]	0.11	0.88[0.66,1.17]	NA	0.98[0.85,1.14]	0.66

Notes: CI, confidence interval; CRC, colorectal cancer; NA, not available; OR, odds ratio; PC, prostate cancer.

^a^ P value of Q test for assessing heterogeneity.

**Table 3:** Overall and subgroup analysis of the *ADIPOR1* rs7539542(C/G) polymorphism and cancer risk.

Variable	Homozygous model		Heterozygous model		Dominant model		Recessive model		Allele contrast model	
	OR [95%CI]	P^a^	OR [95%CI]	P^a^	OR [95%CI]	P^a^	OR [95%CI]	P^a^	OR [95%CI]	P^a^
Total	1.02[0.88,1.18]	0.66	1.05[0.94,1.18]	0.27	1.05[0.95,1.16]	0.43	0.98[0.88,1.10]	0.79	1.01[0.95,1.08]	0.49
*Ethnicity*										
Non-Asian	1.07[0.91,1.25]	0.95	1.08[0.96,1.22]	0.43	1.08[0.97,1.19]	0.72	1.00[0.89,1.13]	0.77	1.04[0.96,1.11]	0.72
Asian	0.74[0.49,1.09]	NA	0.77[0.53,1.14]	NA	0.76[0.52,1.09]	NA	0.89[0.69,1.17]	NA	0.88[0.73,1.06]	NA
*Tumor type*										
CRC	0.93[0.72,1.21]	0.26	0.97[0.70,1.36]	0.06	0.97[0.70,1.35]	0.05	0.96[0.78,1.18]	0.73	1.00[0.89,1.13]	0.12
PC	1.00[0.80,1.24]	1.00	1.13[0.94,1.36]	0.69	1.09[0.92,1.30]	0.68	0.95[0.82,1.09]	0.97	1.00[0.91,1.11]	0.68
Others	1.19[0.88,1.61]	NA	0.96[0.77,1.19]	NA	1.03[0.89,1.20]	0.80	1.21[0.91,1.60]	NA	1.06[0.92,1.23]	NA

Notes: CI, confidence interval; CRC, colorectal cancer; NA not available; OR, odds ratio; PC, prostate cancer.

^a^ P value of Q test for assessing heterogeneity.

**Fig 3:**
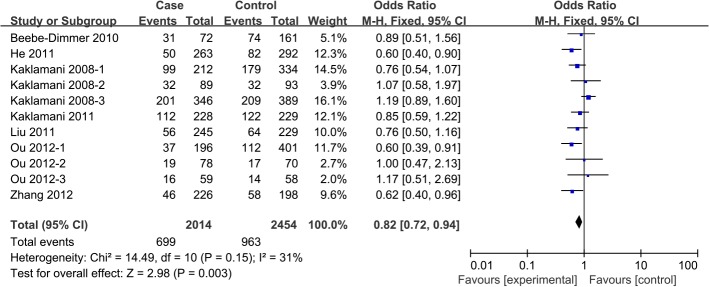
Forest plot of the association between *ADIPOR1* SNP rs1342387(G/A) and cancer risk in a homozygous model.

**Fig 4:**
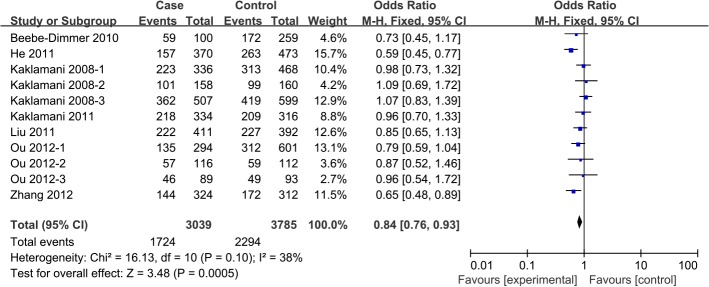
Forest plot of the association between *ADIPOR1* SNP rs1342387(G/A) and cancer risk in a heterozygous model.

In pooled analysis from all 12 studies[11–17, 22, 23], a significant association was observed betweenrs1342387(G/A) and cancer risk, according to four genetic models: homozygous (AA vs. GG, OR 0.82, 95%CI 0.72 to 0.94, P_heterogeneity_ = 0.15), heterozygous (AG vs. GG, OR 0.84, 95%CI 0.76 to 0.93, P_heterogeneity_ = 0.10), dominant (AA+AG vs. GG, OR 0.85, 95%CI 0.75 to 0.97, P_heterogeneity_ = 0.02) and allele contrast (A carriers vs. G carriers, OR 0.88, 95%CI 0.80 to 0.97, P_heterogeneity_ = 0.02) (**Table 4, Figs 3–4**).

**Table 4:** Overall and subgroup analysis of the *ADIPOR1* rs1342387(G/A)polymorphism and cancer risk.

Variable	Homozygous model		Heterozygous model		Dominant model		Recessive model		Allele contrast model	
	OR [95%CI]	P^a^	OR [95%CI]	P^a^	OR [95%CI]	P^a^	OR [95%CI]	P^a^	OR [95%CI]	P^a^
Total	**0.82[0.72,0.94]**	0.15	**0.84[0.76,0.93]**	0.10	**0.85[0.75,0.97]**	0.02	0.90[0.80,1.00]	0.43	**0.88[0.80,0.97]**	0.02
*Ethnicity*										
Non-Asian	0.95[0.80,1.13]	0.34	0.99[0.85,1.15]	0.87	1.00[0.88,1.13]	0.66	0.96[0.84,1.11]	0.27	0.98[0.90,1.06]	0.31
Asian	**0.68[0.56,0.83]**	0.57	**0.74[0.64,0.84]**	0.34	**0.72[0.63,0.82]**	0.28	0**.80[0.67,0.96]**	0.77	**0.79[0.72,0.87]**	0.29
*Tumor type*										
CRC	**0.70[0.59,0.83]**	0.60	**0.79[0.66,0.94]**	0.07	**0.75[0.67,0.84]**	0.10	**0.78[0.67,0.91]**	0.91	**0.81[0.75,0.88]**	0.19
PC	0.86[0.63,1.17]	0.89	0.88[0.67,1.15]	0.34	0.87[0.68,1.12]	0.54	0.92[0.72,1.19]	0.44	0.92[0.79,1.08]	0.95
Others	1.17[0.90,1.51]	0.92	1.02[0.82,1.26]	0.76	1.06[0.91,1.24]	0.89	1.14[0.92,1.40]	0.98	1.07[0.94,1.21]	0.81

Notes: CI, confidence interval; CRC, colorectal cancer; OR, odds ratio; PC, prostate cancer.

^a^ P value of Q test for assessing heterogeneity.

Bold values indicate significant associations.

The association between rs1342387(G/A) and cancer risk was checked in stratified analyses based on ethnicity (**Table 4**). The polymorphism was associated with decreased cancer risk in Asians according to all five genetic models: AA vs. GG, OR 0.68, 95%CI 0.56 to 0.83, P_heterogeneity_ = 0.57; AG vs.GG, OR 0.74, 95%CI 0.64 to 0.84, P_heterogeneity_ = 0.34; AA+AG vs. GG, OR 0.72, 95%CI 0.63 to 0.82, P_heterogeneity_ = 0.28; AA vs. AG+GG, OR 0.80, 95%CI 0.67 to 0.96, P_heterogeneity_ = 0.77; A carriers vs. G carriers, OR 0.79, 95%CI 0.72 to 0.87, P_heterogeneity_ = 0.29. However, no significant association was found in non-Asians.

Next, the association between rs1342387(G/A) and cancer risk was checked in stratified analyses based on cancer type (**Table 4**). The SNP was significantly associated with decreased risk of colorectal cancer, according to all five genetic models: AA vs. GG, OR 0.70, 95%CI 0.59 to 0.83, P_heterogeneity_ = 0.60; AG vs. GG, OR 0.79, 95%CI 0.66 to 0.94, P_heterogeneity_ = 0.07; AA+AG vs. GG, OR 0.75, 95%CI 0.67 to 0.84, P_heterogeneity_ = 0.10; AA vs. AG+GG, OR 0.78, 95%CI 0.67 to 0.91, P_heterogeneity_ = 0.91; A carriers vs. G carriers, OR 0.81, 95%CI 0.75 to 0.88, P_heterogeneity_ = 0.19. However, no significant association was observed for prostate or other cancers.

Besides, the association between rs1342387(G/A) and cancer risk in Asians was also checked in stratified analyses based on cancer type (**Table 5**). The SNP was significantly associated with decreased risk of colorectal cancer in Asians, according to all five genetic models: AA vs. GG, OR 0.64, 95%CI 0.52 to 0.79, P_heterogeneity_ = 0.82; AG vs. GG, OR 0.71, 95%CI 0.62 to 0.82, P_heterogeneity_ = 0.23; AA+AG vs. GG, OR 0.70, 95%CI 0.61 to 0.80, P_heterogeneity_ = 0.28; AA vs. AG+GG, OR 0.76, 95%CI 0.63 to 0.93, P_heterogeneity_ = 0.90; A carriers vs. G carriers, OR 0.77, 95%CI 0.70 to 0.85, P_heterogeneity_ = 0.44, but no significant association was observed for other cancers.

**Table 5:** Overall and subgroup analysis of the *ADIPOR1* rs1342387(G/A)polymorphism and cancer risk in Asians.

Variable	Homozygous model		Heterozygous model		Dominant model		Recessive model		Allele contrast model	
	OR [95%CI]	P^a^	OR [95%CI]	P^a^	OR [95%CI]	P^a^	OR [95%CI]	P^a^	OR [95%CI]	P^a^
Total	**0.68[0.56,0.83]**	0.57	**0.74[0.64,0.84]**	0.34	**0.72[0.63,0.82]**	0.28	0**.80[0.67,0.96]**	0.77	**0.79[0.72,0.87]**	0.29
*Tumor type*										
CRC	**0.64[0.52,0.79]**	0.82	**0.71[0.62,0.82]**	0.23	**0.70[0.61,0.80]**	0.28	**0.76[0.63,0.93]**	0.90	**0.77[0.70,0.85]**	0.44
Others	1.08[0.62,1.88]	0.79	0.91[0.62,1.34]	0.80	0.94[0.66,1.36]	0.76	1.13[0.67,1.90]	0.85	1.00[0.77,1.31]	0.75

Notes: CI, confidence interval; CRC, colorectal cancer; OR, odds ratio.

^a^ P value of Q test for assessing heterogeneity.

Bold values indicate significant associations.

### Sensitivity analysis

**Fig 5:**
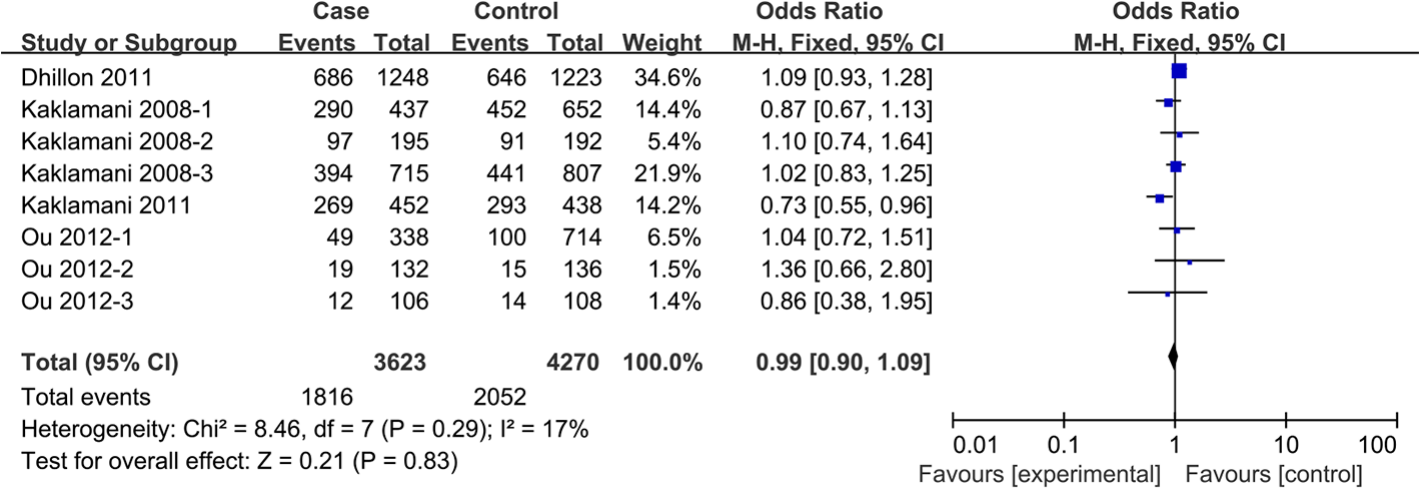
Forest plot of the association between *ADIPOR1* SNP rs12733285(C/T) and cancer risk in a dominant model after sensitivity analysis.

Sensitivity analysis was performed to confirm the main source of heterogeneity across studies. Data for rs12733285(C/T) pooled from all studies showed significant heterogeneity in all genetic models except the recessive model (**Table 2**). Sensitivity analysis identified the primary sources of heterogeneity to be Kaklamani et al. [14] in the homozygous model, He et al. [11] in the heterozygous model, and He et al. [11] and Zhang et al. [12] in both the dominant and allele contrast models. Removing these studies did not significantly alter the results: TT vs.CC, OR 1.03, 95%CI 0.87 to 1.22, P_heterogeneity_ = 0.45; CT vs.CC, OR 0.97, 95%CI 0.88 to 1.07, P_heterogeneity_ = 0.22; TT+CT vs. CC, OR 0.99, 95%CI 0.90 to 1.09, P_heterogeneity_ = 0.29 (**Fig 5**); A carriers vs. G carriers, OR 0.98, 95%CI 0.92 to 1.06, P_heterogeneity_ = 0.12.

**Fig 6:**
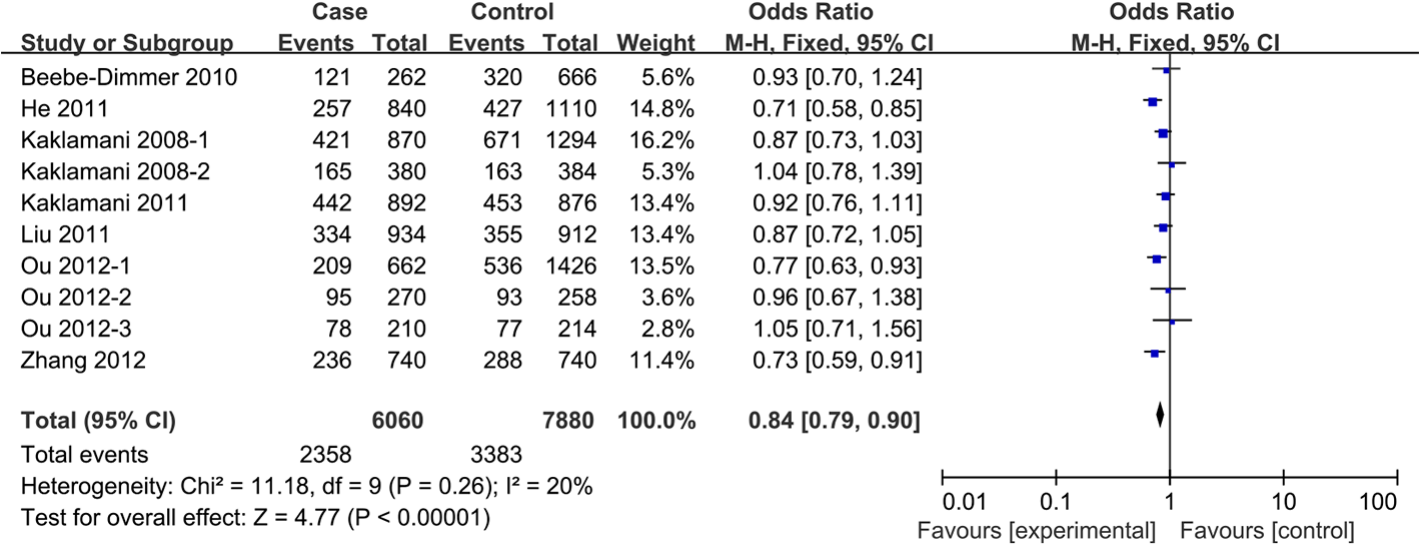
Forest plot of the association between *ADIPOR1* SNP rs1342387(G/A) and cancer risk in an allele contrast model after sensitivity analysis.

Data for rs1342387(G/A) pooled from all studies showed significant heterogeneity in the dominant model, due primarily to He et al. [11], as well as in the allele contrast model, due primarily to Kaklamani et al. [13]. Omitting these studies did not influence the results in the allele contrast model (T carriers vs. G carriers, OR 0.84, 95%CI 0.79 to 0.90, P_heterogeneity_ = 0.26) (**Fig 6**), although it did uncover a borderline association in the dominant model (AA+AG vs. GG, OR 0.89, 95%CI 0.80 to 1.00, P_heterogeneity_ = 0.18).

### Publication bias

**Fig 7:**
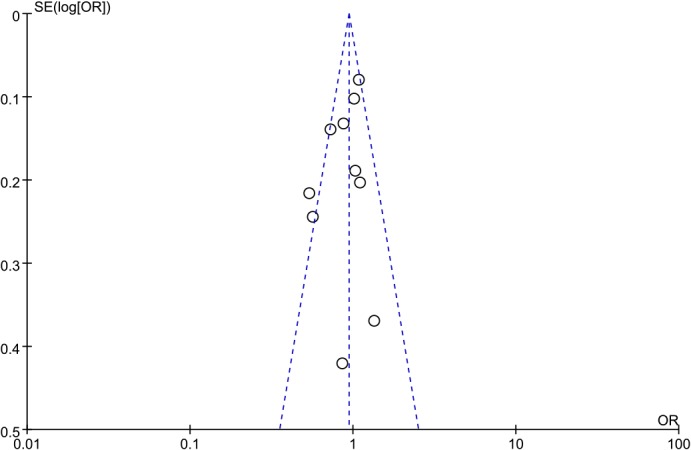
Funnel plot to detect publication bias in data on *ADIPOR1* SNP rs12733285(C/T) according to a dominant model.

**Fig 8:**
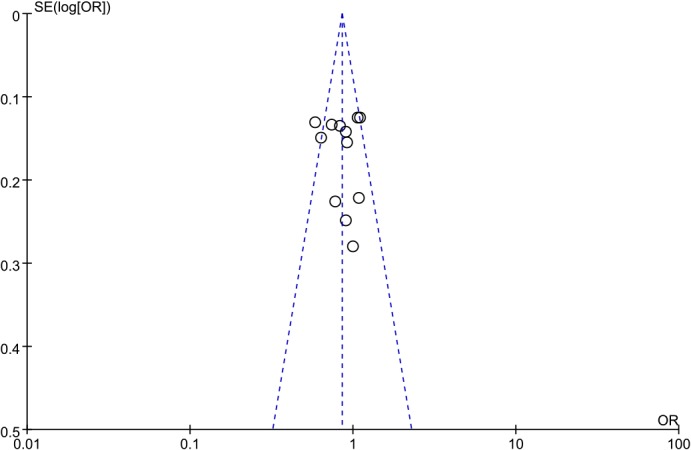
Funnel plot to detect publication bias in data on *ADIPOR1* SNP rs1342387(G/A) according to a dominant model.

**Fig 9:**
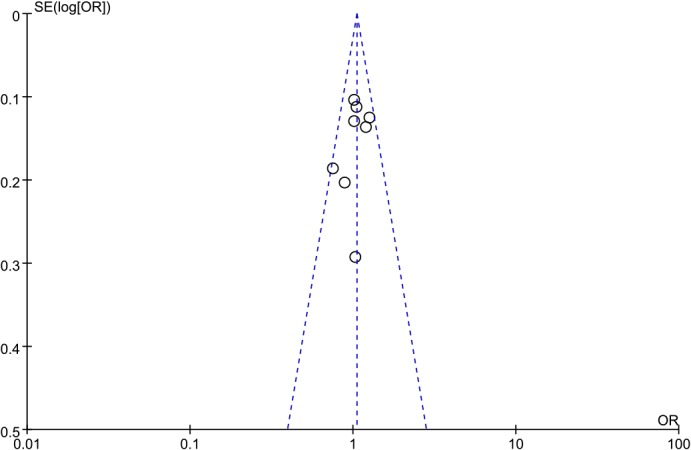
Funnel plot to detect publication bias in data on *ADIPOR1* SNP rs7539542(C/G) according to a dominant model.

Visual inspection of the funnel plots (**Figs 7–9**) suggested a roughly symmetrical distribution for the studies covering each of the *ADIPOR1* SNPs according to the dominant model, indicating low risk of publication bias in the meta-analysis. Similarly, Egger’s and Begg’s tests revealed no significant potential for publication bias under the dominant model: rs12733285(C/T), P_Begg_ = 0.325 and P_Egger_ = 0.252; rs1342387(G/A), P_Begg_ = 0.784 and P_Egger_ = 0.785; and rs7539542(C/G), P_Begg_ = 0.621 and P_Egger_ = 0.368.

## Discussion

ADIPOR1, expressed at high levels in skeletal muscle and pancreatic beta cells [24–26], is expressed in many types of cancer, including breast, colorectal, pancreatic, and esophageal cancers [27–30]. Despite numerous studies of the possible association of *ADIPOR1* SNPs rs12733285(C/T), rs1342387(G/A) and rs7539542(C/G) with cancer risk [10–17, 22, 23], whether these polymorphisms are indeed associated with cancer risk remains unclear. Combining the statistical power of 13 case-control studies in this meta-analysis, we show that the A allele of *ADIPOR1* rs1342387 is associated with significantly lower risk of colorectal cancer than is the G allele in Asians, suggesting that the A allele may protect against such cancer in this ethnic group. This SNP does not appear to be associated with risk of other cancers in Asians, or with risk of any cancers in non-Asians. The SNPs rs12733285(C/T) and rs7539542(C/G) did not show significant associations with any type of cancer in meta-analyses involving all data or data from subgroups.

To verify the reliability of our meta-analyses, we performed sensitivity analyses when significant heterogeneity was present across pooled studies. Removing the studies that explained most of this heterogeneity did not significantly alter the initial results, confirming their reliability. We also sought to reduce publication bias by searching not only in Western databases of research literature but also in the major Chinese ones. Studies have shown that for some areas of genetic epidemiology, Chinese-language journals not indexed in PubMed contain a higher proportion of articles reporting nonsignificant results than do PubMed-indexed journals [31], so combining Western and Chinese databases may help us to reduce selective reporting bias.

Our results suggesting that the A allele of rs1342387(G/A) protects against colorectal cancer at least in Asians is consistent with a previous report that the A allele is associated with higher serum levels of adiponectin [32], and serum adiponectin levels are inversely associated with risk of obesity-related malignancies [5–7]. One study reported an association of rs1342387(G/A)with increased colorectal cancer risk in a single Caucasian population using the dominant model [13], but the association disappeared upon re-analysis using a Cockerham model [33].Similarly, logistic regression analysis of 7,020 cases and 7,631 controls of European descent failed to find an association between rs1342387(G/A) and risk of colorectal cancer [34]. That these studies failed to detect an association reflects the diverse effects of *ADIPOR1* variants in Caucasians, consistent with the present study. ADIPOR1 and ADIPOR2 mediate the link between adiponectin and activation of AMP-activated protein kinase, which causes adiponectin to exert anti-proliferative effects under cancer conditions [8].The *ADIPOR1* SNP rs1342387(G/A)may modulate the effects of adiponectin on cancer risk by regulating the expression of adiponectin receptors, but our results suggest that this is not necessarily true in all cancers and all ethnicities. This may help explain conflicting reports in the literature about the association of this SNP with cancer risk.

Our findings that the A allele of rs1342387 protects against colorectal cancer in Asians and that rs12733285(C/T) shows no significant associations with colorectal cancer risk were also reported in a meta-analysis by Ou et al. [23]. The present work extends that study in several important ways. First, we included a larger number of colorectal cancer patients than Ou et al. Second, we performed subgroup analyses based on ethnicity and cancer type, while Ou et al. did not. Our findings are therefore a critical contribution to the literature because they provide strong evidence that the same *ADIPOR1* SNP can exert more or less influence on cancer risk depending on the type of cancer and ethnicity. Third, we examined associations between the SNPs and cancer risk using five genetic models, whereas Ou et al. reported results using only the dominant model.

Similar to the present meta-analysis, Yu et al. reported in their meta-analysis that the SNP rs1342387(G/A) is associated with colorectal cancer risk in Asians [35]. Our study extends those findings, because Yu et al. did not use as large a sample size as we did, nor did they examine relationships betweenrs12733285(C/T) or rs7539542(C/G) and risk of cancer. In addition, Yu et al. did not checked the association between rs1342387(G/A) and cancer risk in Asians based on stratified analyses.

Despite its strengths, our meta-analysis has several limitations. First, it focused only on SNPs, but numerous factors act individually and together to influence risk of cancer, including lifestyle, dietary habits, environment, and genetics. The included studies in our meta-analysis reported data on few or none of these issues, making it impossible for us to assess them across patients and controls. Second, since various types of cancer were included, the patient and control populations were heterogeneous. The different sources of controls (population- or hospital-based) might create selection bias toward the null hypothesis. Third, the meta-analysis included a relatively small number of studies and did not take into account unpublished data or “grey literature”. This may raise the risk of publication bias, even though our analyses suggest the absence of significant risk.

In conclusion, our meta-analysis suggests that the *ADIPOR1* SNP rs1342387(G/A), but not the SNPs rs12733285(C/T) or rs7539542(C/G), are associated with cancer risk, especially risk of colorectal cancer in Asians. Large, well-designed studies are needed to verify and extend our findings.

## Supporting Information

S1 Checklist PRISMA 2009 checklist. (DOC)

S2 Checklist Meta-analysis of Genetic Association Studies checklist. (DOC)

S1 Appendix The 21 excluded articles and the reasons. (DOC)

S1 Table Results of quality assessment using the Newcastle–Ottawa Scale for case-control studies. (DOC)
