# Supplementary material for: Three ADIPOR1 Polymorphisms and Cancer Risk: A Meta-Analysis of Case-Control Studies
Source: PLoS One. 2015 Jun 5;10(6):e0127253. doi: 10.1371/journal.pone.0127253 (PMC4457489; doi:10.1371/journal.pone.0127253)
Supplement: S1 Appendix — (DOC) [file pone.0127253.s003.doc]

The 21 excluded articles and the reasons

1. Li Q, Ma Y, Sang W, Cui W, Li X, et al. (2014) Five Common Haplotype-Tagging Variants of Adiponectin (ADIPOQ) and Cancer Susceptibility: A Meta-Analysis. Genetic testing and molecular biomarkers 18: 417-424.

2. Li P, Liu H, Li C, Yang B, Kong Q, et al. (2014) An updated meta-analysis of the association between ADIPOQ rs2241766 polymorphism and colorectal cancer. Tumour biology : the journal of the International Society for Oncodevelopmental Biology and Medicine 35: 2491-2496.

3. Zhou W, Liu Y, Zhong DW (2013) Adiponectin (ADIPOQ) rs2241766 G/T polymorphism is associated with risk of cancer: evidence from a meta-analysis. Tumour biology : the journal of the International Society for Oncodevelopmental Biology and Medicine 34: 493-504.

4. Ye CC, Tan SY, Wang J, Li M, Zhang J, et al. (2013) Association between adiponectin gene polymorphisms and risk of colorectal cancer: A Metaanalysis. World Chinese Journal of Digestology 21: 3030-3036.

5. Ye C, Wang J, Tan S, Zhang J, Li M, et al. (2013) Meta-analysis of adiponectin polymorphisms and colorectal cancer risk. International journal of medical sciences 10: 1113-1120.

6. Yang Y, Zhang F, Ding R, Skrip L, Wang Y, et al. (2013) ADIPOQ gene polymorphisms and cancer risk: a meta-analysis. Cytokine 61: 565-571.

7. Xu Y, He B, Pan Y, Gu L, Nie Z, et al. (2013) The roles of ADIPOQ genetic variations in cancer risk: evidence from published studies. Molecular biology reports 40: 1135-1144.

8. VanSaun MN (2013) Molecular pathways: Adiponectin and leptin signaling in cancer. Clinical Cancer Research 19: 1926-1932.

9. Kaklamani VG, Hoffmann TJ, Thornton TA, Hayes G, Chlebowski R, et al. (2013) Adiponectin pathway polymorphisms and risk of breast cancer in African Americans and Hispanics in the Women's Health Initiative. Breast cancer research and treatment 139: 461-468.

10. Fan HJ, Wen ZF, Xu BL, Wu JJ, Jia YX, et al. (2013) Three adiponectin rs1501299G/T, rs822395A/C, and rs822396A/G polymorphisms and risk of cancer development: a meta-analysis. Tumour biology : the journal of the International Society for Oncodevelopmental Biology and Medicine 34: 769-778.

11. Nock NL, Levine A, Neslund-Dudas C, Beebe-Dimmer J, Bock C, et al. (2012) Integrating multiple genetic and environmental factors using structural equation modeling: An application to obesity, adipokine and cytokine signaling pathways and prostate cancer risk. Genetic Epidemiology 36: 144-145.

12. Dalamaga M, Diakopoulos KN, Mantzoros CS (2012) The role of adiponectin in cancer: A review of current evidence. Endocrine Reviews 33: 547-594.

13. An W, Bai Y, Deng SX, Gao J, Ben QW, et al. (2012) Adiponectin levels in patients with colorectal cancer and adenoma: A meta-analysis. European Journal of Cancer Prevention 21: 126-133.

14. Yi N, Kaklamani VG, Pasche B (2011) Bayesian analysis of genetic interactions in case-control studies, with application to adiponectin genes and colorectal cancer risk. Annals of Human Genetics 75: 90-104.

15. Stark JR, Finn SP, Ma J, Sinnott JA, Schumacher F, et al. (2011) Adiponectin receptor 2 expression predicts lethal prostate cancer. Laboratory Investigation 91: 226A.

16. Enns JE, Taylor CG, Zahradka P (2011) Variations in Adipokine Genes AdipoQ, Lep, and LepR are Associated with Risk for Obesity-Related Metabolic Disease: The Modulatory Role of Gene-Nutrient Interactions. Journal of obesity 2011: 168659.

17. Conroy SM, Cheng I, Caberto CP, Tiirikainen M, Kolonel L, et al. (2011) A comprehensive analysis of common genetic variation in adiponectin with colorectal cancer and biomarkers of insulin resistance: The multiethnic cohort. American Journal of Epidemiology 173: S18.

18. Klein F (2009) Colorectal cancer: Slight risk in variant of the adiponectin gene. Zeitschrift fur Gastroenterologie 47: 186.

19. Baillargeon J, Rose DP (2006) Obesity, adipokines, and prostate cancer (review). International journal of oncology 28: 737-745.

Reason: The nineteen articles above is not case-control study.

1. Ye L, Zhang ZY, Du WD, Schneider ME, Qiu Y, et al. (2013) Genetic analysis of ADIPOQ variants and gastric cancer risk: a hospital-based case-control study in China. Medical oncology (Northwood, London, England) 30: 658.

2. Chen X, Xiang YB, Long JR, Cai H, Cai Q, et al. (2012) Genetic polymorphisms in obesity-related genes and endometrial cancer risk. Cancer 118: 3356-3364.

Reason: The two articles above lack of available data.
