# Supplementary material for: Three ADIPOR1 Polymorphisms and Cancer Risk: A Meta-Analysis of Case-Control Studies
Source: PLoS One. 2015 Jun 5;10(6):e0127253. doi: 10.1371/journal.pone.0127253 (PMC4457489; doi:10.1371/journal.pone.0127253)
Supplement: S1 Table — (DOC) [file pone.0127253.s004.doc]

**Table S1.** Results of quality assessment using the Newcastle–Ottawa Scale for case-control studies

| Study | Selection |  |  |  | Comparability | Exposure |  |  | Scores |
| --- | --- | --- | --- | --- | --- | --- | --- | --- | --- |
|  | Adequate  definition of cases | Representat-  iveness of  the cases | Selection of con-  trols | Definition of con-  trols | Control for  important factor ^a^ | Ascertain-  ment of  exposure | Same method  of ascertain-  ment for cases  and controls | Non-  response  rate |  |
| Dhillon 2011[16] | **☆** | **☆** | **☆** | **☆** | **☆☆** | **☆** | **☆** | **☆** | 9 |
| He 2011[11] | **☆** | **☆** | **-** | **☆** | **☆☆** | **☆** | **☆** | **-** | 7 |
| Kaklamani 2008-1[13] | **☆** | **☆** | **☆** | **☆** | **☆** | **☆** | **☆** | **☆** | 8 |
| Kaklamani 2008-1[13] | **☆** | **☆** | **-** | **☆** | **☆☆** | **☆** | **☆** | **☆** | 8 |
| Kaklamani 2008-3[18] | **☆** | **☆** | **☆** | **☆** | **☆** | **☆** | **☆** | **-** | 7 |
| Kaklamani 2011[14] | **☆** | **☆** | **☆** | **☆** | **☆☆** | **☆** | **☆** | **-** | 8 |
| Ou 2012-1[23] | **☆** | **☆** | **-** | **☆** | **☆** | **☆** | **☆** | **-** | 6 |
| Ou 2012-2[23] | **☆** | **☆** | **☆** | **☆** | **☆☆** | **☆** | **☆** | **-** | 8 |
| Ou 2012-2[23] | **☆** | **☆** | **☆** | **☆** | **☆☆** | **☆** | **☆** | **-** | 8 |
| Zhang 2012[12] | **☆** | **☆** | **-** | **☆** | **☆☆** | **☆** | **☆** | **-** | 7 |
| Beebe-  Dimmer 2010[15] | **☆** | **☆** | **☆** | **-** | **☆** | **☆** | **☆** | **-** | 6 |
| Liu 2011[24] | **☆** | **☆** | **-** | **☆** | **☆☆** | **☆** | **☆** | **-** | 7 |
| Teras 2009[17] | **☆** | **☆** | **☆** | **☆** | **☆☆** | **☆** | **☆** | **-** | 8 |

^a^ A maximum of 2 stars can be allotted in this category, one for age, the other for other controlled factors.

**NEWCASTLE - OTTAWA QUALITY ASSESSMENT SCALE**

**FOR CASE CONTROL STUDIES**

Note: A study can be awarded a maximum of one star for each numbered item within the Selection and Exposure categories. A maximum of two stars can be given for Comparability

**Selection**

1) Is the case definition adequate?

a) yes, with independent validation **🟑**

b) yes, eg record linkage or based on self reports

c) no description

2) Representativeness of the cases

a) consecutive or obviously representative series of cases **🟑**

b) potential for selection biases or not stated

3) Selection of Controls

a) community controls **🟑**

b) hospital controls

c) no description

4) Definition of Controls

a) no history of disease (endpoint) **🟑**

b) no description of source

**Comparability**

1) Comparability of cases and controls on the basis of the design or analysis

a) study controls for _______________ (Select the most important factor.) **🟑**

b) study controls for any additional factor **🟑**(This criteria could be modified to indicate specific control for a second important factor.)

**Exposure**

1) Ascertainment of exposure

a) secure record (eg surgical records) **🟑**

b) structured interview where blind to case/control status **🟑**

c) interview not blinded to case/control status

d) written self report or medical record only

e) no description

2) Same method of ascertainment for cases and controls

a) yes **🟑**

b) no

3) Non-Response rate

a) same rate for both groups **🟑**

b) non respondents described

c) rate different and no designation
